# Spatial assessments in texture analysis: what the radiologist needs to know

**DOI:** 10.3389/fradi.2023.1240544

**Published:** 2023-08-24

**Authors:** Bino A. Varghese, Brandon K. K. Fields, Darryl H. Hwang, Vinay A. Duddalwar, George R. Matcuk, Steven Y. Cen

**Affiliations:** ^1^ Department of Radiology, Keck School of Medicine, University of Southern California, Los Angeles, CA, United States; ^2^ Department of Radiology & Biomedical Imaging, University of California, San Francisco, San Francisco, CA, United States; ^3^ Department of Radiology, Cedars-Sinai Medical Center, Los Angeles, CA, United States

**Keywords:** radiomics, texture analysis, spatial assessment, machine learning, artificial intelligence

## Abstract

To date, studies investigating radiomics-based predictive models have tended to err on the side of data-driven or exploratory analysis of many thousands of extracted features. In particular, spatial assessments of texture have proven to be especially adept at assessing for features of intratumoral heterogeneity in oncologic imaging, which likewise may correspond with tumor biology and behavior. These spatial assessments can be generally classified as spatial filters, which detect areas of rapid change within the grayscale in order to enhance edges and/or textures within an image, or neighborhood-based methods, which quantify gray-level differences of neighboring pixels/voxels within a set distance. Given the high dimensionality of radiomics datasets, data dimensionality reduction methods have been proposed in an attempt to optimize model performance in machine learning studies; however, it should be noted that these approaches should only be applied to training data in order to avoid information leakage and model overfitting. While area under the curve of the receiver operating characteristic is perhaps the most commonly reported assessment of model performance, it is prone to overestimation when output classifications are unbalanced. In such cases, confusion matrices may be additionally reported, whereby diagnostic cut points for model predicted probability may hold more clinical significance to clinical colleagues with respect to related forms of diagnostic testing.

## Key points

• Features of intratumoral heterogeneity are well-represented by spatial assessments of texture, which may similarly correlate with tumor biology and behavior.
• Spatial filters are used to enhance edges and/or textures of an image by identifying areas of rapid change within the grayscale.
• Neighborhood-based methods are higher-order texture approaches which quantify differences in gray-level intensities of particular regions of interest with respect to their neighbors within a set distance.

## Introduction

Quantitative assessments of imaging texture characteristics have been successfully applied to answer a variety of clinically-relevant queries ranging from lesion classification to disease prognostication, often in the form of radiomics-based machine learning decision classifiers (1–13). While some approaches have previously relied on filtering of high-dimensionality data to identify the most contributory features or classes of features (14–17), recent studies have demonstrated a subset of texture metrics well-equipped to detect regions of heterogeneity in the imaging grayscale (4, 9) (Supplementary Table S1). These “spatial assessments” are aptly named for their ability to resolve subtle areas of voxel-to-voxel variation, or in plainer terms, what might be subjectively referred to as “coarseness” by a human interpreting radiologist (4, 7, 8, 18–23). In this review, we detail the various common approaches to spatial assessment of imaging texture, as well as their applicability and implications in future radiomics and machine learning-related studies.

## Approaches to spatial assessment

### Spatial filters

Spatial filters are image processing methods that enhance spatial image properties of a region of interest such as edges and/or textures (23–25). The size and shape of the filter neighborhood or convolution kernel determines the performance of the filter, and warrants standardization across multiple studies to evaluate reliability (26). Some commonly used spatial filters for texture analysis include statistical filters such as entropy filters, range filters, standard deviation filters, median filters, and average filters. However, given that use of spatial filters can lead to an increase in radiomics feature space (27), it is advised to avoid using these approaches with small sample sizes.

Directional gradients and direction invariant gradients have been used to improve edge enhancement. For example, edge filters such as Kirsch and Sobel have been reported as part of multiple radiomics panels (28, 29). Likewise, the Laplacian of Gaussian filter, which captures edges based on detecting areas of rapid change in grayscale intensity and then smooths them with a standard-deviation tunable Gaussian bandpass filter, has been reported frequently in radiomics panels to capture areas with increasingly coarse texture patterns (24, 27, 30). Kernels such as the Laws filters identify specific textures based on five fundamental vectors that emphasize features of edge, level, spot, ripple, and wave, or a combination thereof, and have been used for spatial filtering prior to feature extraction (23, 31).

In some cases, noise can be suppressed using image transforms, such as Fourier analysis (24). In this method, spatial domain information can be converted to frequency domain information and then filtered for high frequencies, low frequencies, bandpass, etc. However, while the signal to noise ratio can be improved, this technique merely suppresses the noise without improving the strength of the underlying signal (32). Wavelet transforms further build upon the Fourier technique by decomposing the original image in both spatial and frequency domains, thereby providing relatively more precise signal localization (24, 27, 33, 34). The coefficients of these decomposed sub-bands can then be weighted to enhance specific signal properties along select directions of a 3-dimensional space.

### Neighborhood-based methods

**Figure 1:**
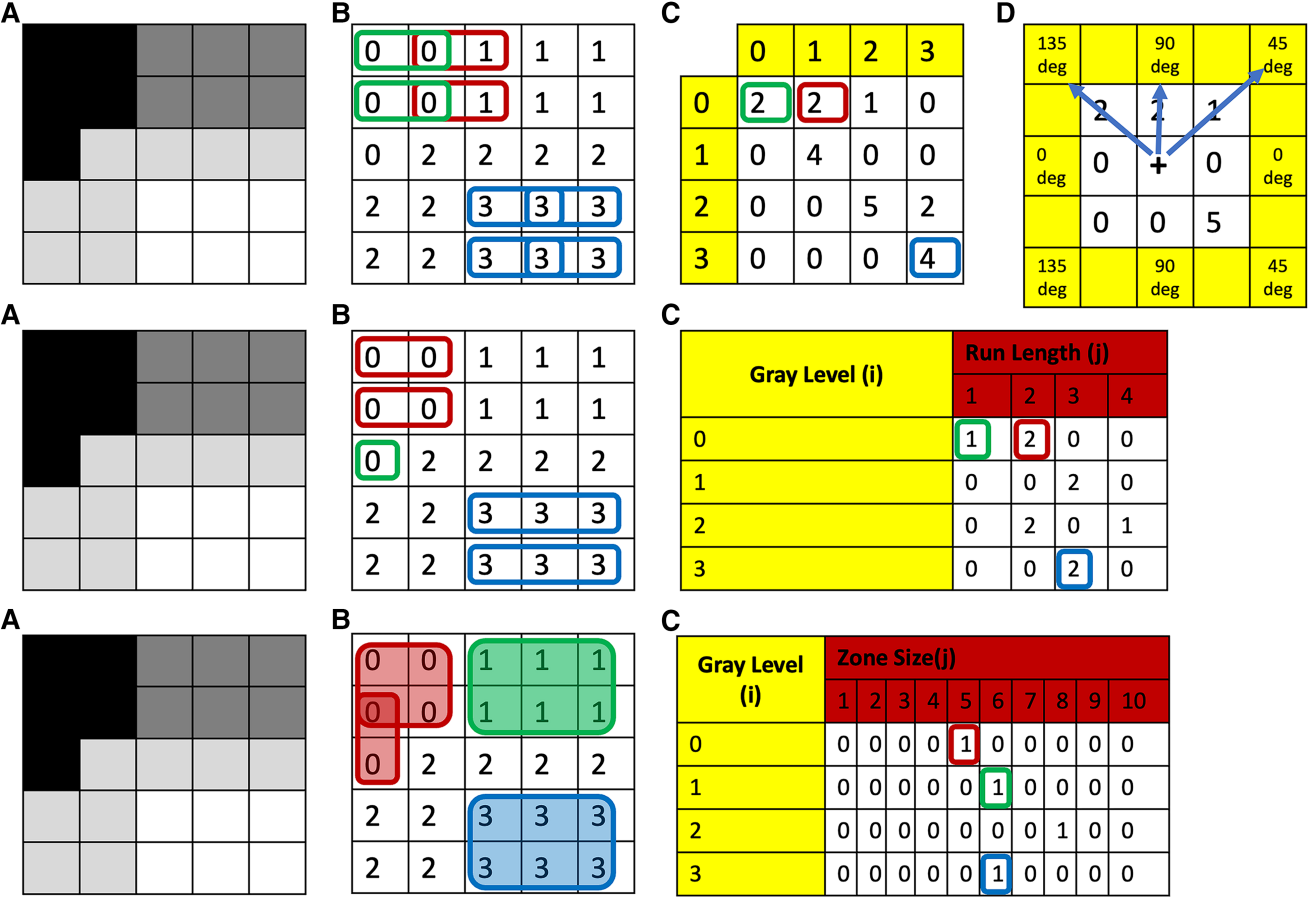
Top row: (**A**) grayscale image with four different gray-levels. (**B**) Digitized version of the gray-level image with unique numerical values corresponding to the gray-level or a range of gray-levels (dependent on bin size of bin width) for each theoretical pixel/voxel. (**C**) GLCM map of the image obtained for distance 1 and direction 0 degrees. (**D**) This same process is then repeated in all other directions: i.e., 45, 90, and 135 deg, respectively. To obtain direction invariant results, all results are normalized and averaged. Middle row: (**A**) Grayscale image with four different gray-levels. (**B**) Digitized version of the gray-level image with unique numerical values corresponding to the gray-level or a range of gray-levels (dependent on bin size of bin width) for each theoretical pixel/voxel. (**C**) GLRLM map of the image obtained for direction zero degrees. This same process is then repeated in all other directions: i.e., 45, 90, and 135 deg, respectively. To obtain direction invariant results all results are normalized and averaged. Bottom row: (**A**) grayscale image with four different gray-levels. (**B**) Digitized version of the gray-level image with unique numerical values corresponding to the gray-level or a range of gray-levels (dependent on bin size of bin width) for each theoretical pixel/voxel. (**C**) GLSZM map of the image. GLCM, Gray-Level Co-Occurrence Matrix; GLRLM, Gray-Level Run-Length Matrix; GLSZM, Gray-Level Size-Zone Matrix.

**Figure 2:**
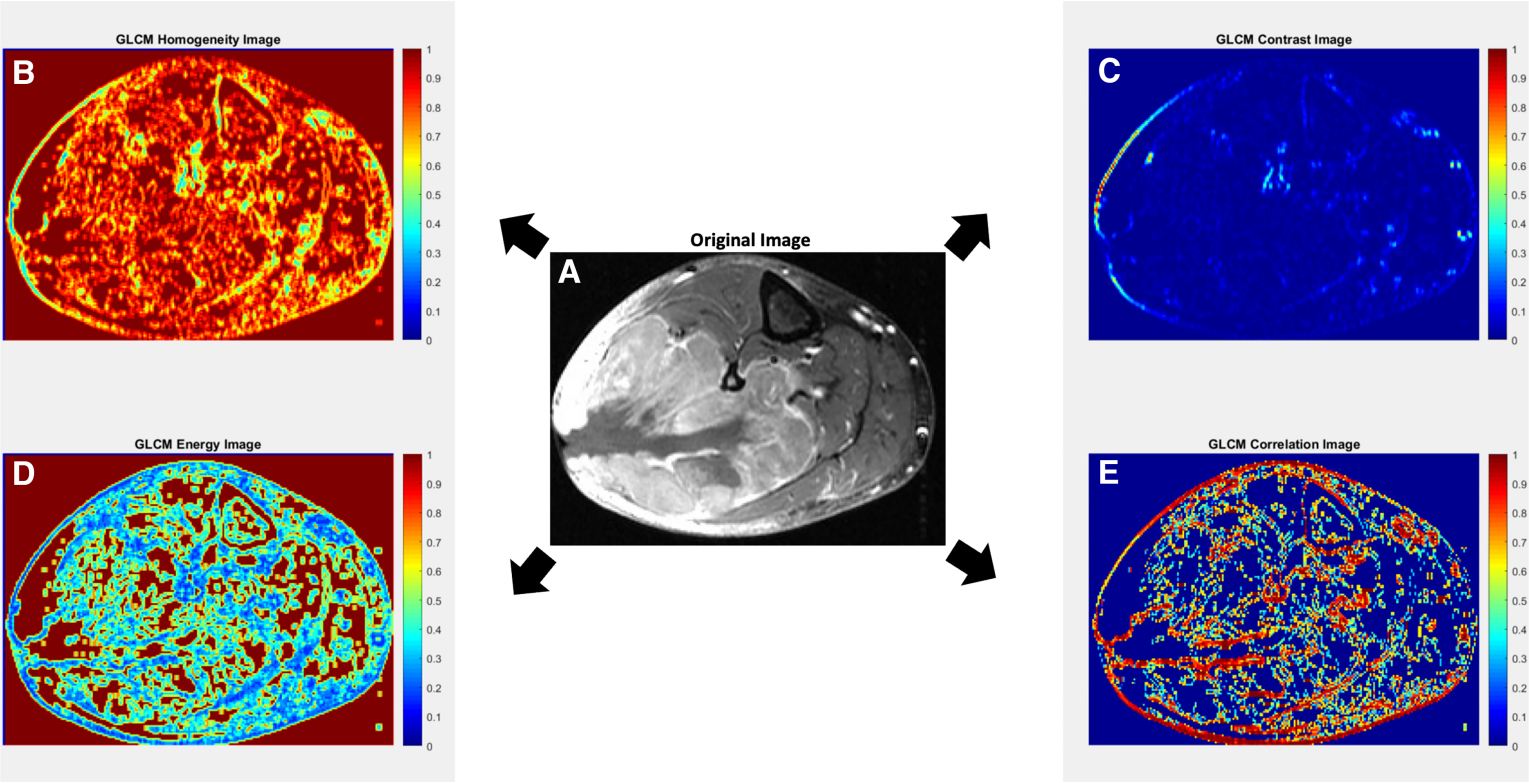
Axial contrast-enhanced T1-weighted MR image with fat suppression of a 70-year-old male with leiomyosarcoma of the posterolateral calf (**A**), with corresponding texture parameter maps for Gray-Level Co-Occurrence Matrix (GLCM) homogeneity (**B**), GLCM contrast (**C**), GLCM energy (**D**) and GLCM correlation (**E**). The GLCM homogeneity map (**B**) reflects the closeness of the distribution of elements in the GLCM map relative to the GLCM diagonal. Highly homogenous regions (i.e., regions with less variation; close to the GLCM diagonal) receive a value of 1, while highly heterogenous regions receive a value of 0. The GLCM contrast map (**C**) measures the intensity contrast between an index pixel and its neighborhood pixels. Regions of high contrast show high heterogeneity in values up to a maximum value of 1. A constant image receives a value of 0. In some studies, contrast may also be referred to as variance and inertia. The GLCM energy map (**D**) measures the sum of the squared elements in the GLCM, whereby highly homogenous regions receive values of 1 and highly heterogenous regions receive values of 0. In some studies, energy may also be referred to as angular second moment, uniformity, or uniformity of energy. The GLCM correlation map (**E**) reflects how correlated a given pixel is to its neighboring pixels, with highly correlated regions receiving values of 1. In general, a neighborhood of 3 × 3 was adopted for the GLCM approach. Original image (**A**) courtesy of The Cancer Genome Atlas Sarcoma Collection (TCGA-SARC) based on data generated by the TCGA research network: http://cancergenome.nih.gov/ (38, 39).

Statistical characterizations of texture can also be assessed from higher-order texture methods (i.e., analysis based on both grayscale values and their spatial orientation) such as Gray-Level Co-Occurrence Matrix (GLCM), Gray-Level Run-Length Matrix (GLRLM), Gray-Level Size-Zone Matrix (GLSZM), Gray-Level Dependence Matrix (GLDM) and Neighborhood Gray-Tone Difference Matrix (NGTDM) (35, 36). In all of these methods, the metrics generated essentially quantify the differences in grayscale brightness between neighboring pixels/voxels (9, 27, 37). For example, in GLCM, texture is quantified based on how often a combination of gray-level values occur next to each other at a given distance and direction within a region of interest (23, 27, 31, 37) (Figure 1, top row). Some commonly reported GLCM metrics include energy, contrast, entropy, homogeneity, correlation, variance, sum average, and autocorrelation (9, 35, 36) (Figure 2).

In contrast to GLCM, GLRLM quantifies the pattern of gray-level intensity pixels in a fixed direction from an interference pixel (Figure 1, middle row). Run-length is defined as the number of adjacent pixels that have the same gray-level intensity in each direction (37). Some commonly reported GLRLM metrics include short and long run emphasis, gray-level non-uniformity, run-length non uniformity, low and high gray-level run emphasis, and their combinations (9, 35).

Similar to GLCM, in GLSZM texture is also quantified based on how often a combination of gray-level values occurs next to each other at a given distance within a region of interest (27, 37); however, in contrast to GLCM, GLSZM is direction independent (40) (Figure 1, bottom row). Some commonly reported GLSZM metrics include short and long zone emphasis, gray-level non-uniformity, zone-size non-uniformity, low and high gray-level zone emphasis, and their combinations (9, 35).

Likewise, GLDM quantifies the number of connected voxels within a set distance that are dependent on a center voxel (37). A neighboring voxel is considered dependent on the center voxel if the absolute difference of their respective gray-levels falls within a set value (9, 41). Some commonly reported GLDM metrics include short and long dependence emphasis, gray-level non-uniformity, dependence non-uniformity, gray-level and dependence variance, and high grey-level zone emphasis, and their combinations.

Lastly, NGTDM evaluates the difference between a particular gray-level intensity and the average gray-level intensity of its neighborhood within a given distance (23, 37, 42). Some commonly reported NGTDM metrics include busyness, coarseness, contrast, strength, and complexity (9, 35).

### Other approaches

Structural methods involve techniques of decomposing an image into basic units and then identifying the rules required to construct that given image from its basic units. For instance, Fractal Dimension (FD) is a metric that evaluates image complexity by quantifying how changes in image scale affect image detail (9, 43, 44). FD uses self-repeating structural patterns in order to quantitatively assess the homogeneity of the region of interest, and increases with greater geometric complexity (35, 43, 45, 46). This in essence functions as an objective evaluation of how consistent a shape is with itself, and thus serves as an excellent measurement of the regularity of a tumor's morphology (23, 44).

## Applications in radiomics and machine learning

In oncologic imaging, radiomics analysis has shown great utility in evaluating features of intratumoral heterogeneity, which may correspondingly reflect tumor behavior (4, 5, 7–9, 11, 13, 14, 35, 47, 48). There is a growing body of literature to suggest that radiomics-based machine learning algorithms perform well with various classification tasks, including differentiating benign from malignant lesions, stratifying lesions by tumor grade, predicting risk of distant metastases, and predicting overall survival (1–13). Additional work suggests that subtle differences in the underlying texture grayscale may also correlate well with tumoral genetic and phenotypic variations, furthering the case for potential future integrations of radiomics classifiers as risk stratification schema in prospective clinical workflows (31, 35, 49, 50).

Given the sheer number of radiomics features extracted as part of standard pipeline workflows, analyses of radiomics datasets are often necessarily complex and difficult to comprehend. Moreover, segmentation approaches (i.e., manual vs. semi-automated vs. fully-automated) can likewise affect the extracted radiomics parameters and—particularly in the case of manual segmentation—be a source of intra- and inter-observer variability (51, 52). Initial statistical considerations should include descriptive analyses to evaluate for skewness, kurtosis, and outlier detection, which in turn hold implications for the reproducibility of a study (53). Missing data may arise from situations where a given radiomics approach does not yield a numerical value, possibly due to image quality degradation or methodological failure. When working with sufficiently high-quality images, missing radiomics data are rarely encountered; however, missing data become much more prevalent as image quality degrades, and, in such cases, imputation methods will often be inaccurate (54, 55). Given this, we believe best practice is to simply exclude subjects with poor image quality and high numbers of missing radiomics features in order to avoid spurious associations. In cases of random missing phases in multiphase studies, we have found in our own research paradigms that imputation methods, such as the Markov Chain Monte Carlo (MCMC) method, work well given high correlation of radiomics features between contrast phases (56).

Data dimensionality reduction methods have often been described in the literature with both supervised and unsupervised machine learning constructions in an attempt to optimize classifier performance. These approaches mainly include data filtering, principal component analysis (PCA), and elimination of highly correlated features (57). However, if used, dimensionality reduction techniques must only be conducted with the training data in order to avoid information leakage, which can in turn bias the decision classifiers and lead to problems of overfitting (58). For example, PCA often suffers from poor reproducibility when applied to test data because its components are derived to maximize the variance explained in the training data (57, 59). Instead, we recommend that removal of highly correlated data (e.g., redundant features with *r* > 0.8 suggesting collinearity) should be performed as the initial approach for dimensionality reduction (60).

Reporting of machine learning performance for radiomics based models is commonly done using area under the curve (AUC) of the receiver operating characteristic (ROC). In general, while AUC can well-represent overall model prediction accuracies, it is prone to overestimating performance in cases of unbalanced classification outputs. To overcome this, a common approach is to report confusion matrices—including sensitivity (recall), specificity (selectivity), positive predictive value (precision), and negative predictive value—corresponding to various cut points for model predicted probability. These values likewise tend to be more easily understood by clinical colleagues, whereby diagnostic cut points in some ways hold more tangible clinical significance with respect to other forms of diagnostic testing. To obtain optimal cut points, common practice includes statistical approaches such as Youden's J statistic (also referred to as Youden's index), defined as J = sensitivity + specificity—1, or simply selecting the cut points that maximize the product of sensitivity and specificity (61, 62). An arguably more sophisticated approach would be to adapt the concept of decision analysis. Decision analysis includes assessing for clinical value by also considering clinical consequences when making determinations of cut point appropriateness, such as weighing the benefits of finding a malignant tumor against the harms of unnecessary biopsies (63). Finally, reporting of machine learning performance should also highlight the variables of importance (VOIs). VOIs are defined as those metrics which are identified as having the greatest impact on classification accuracy and tend to be the most robust features for predicting the queried clinical outcomes. While different machine learning approaches have different methods for selecting VOIs, many also incorporate some form of ranking procedure based on the relative contribution of each metric or class of metrics. These rankings may in turn be useful for identifying potential correlative relationships between the investigated quantitative imaging features and phenotypic observations of disease state (64, 65).

## Conclusion

Machine learning analyses of radiomics feature sets have been applied to a wide array of classification and prognostication tasks in oncologic imaging. Spatial assessments in particular have shown great potential to quantitatively evaluate features of intratumoral heterogeneity and may one day prove to be important prognostic biomarkers of phenotypic behavior in oncologic care. In this review, we discussed some of the most common approaches to spatial assessment of texture in radiologic imaging as well as familiar reporting metrics to assess model performance in machine learning studies.
