# Supplementary material for: Spatial assessments in texture analysis: what the radiologist needs to know
Source: Front Radiol. 2023 Aug 24;3:1240544. doi: 10.3389/fradi.2023.1240544 (PMC10484588; doi:10.3389/fradi.2023.1240544)
Supplement: Supplementary file 1 [file Table1.docx]

**Supplemental Table S1** Summary of selected studies investigating spatial assessments of imaging texture in oncologic imaging.

| **First Author [Reference no.]** | **Year** | **Spatial Assessments Investigated** | **Clinical Correlate** |
| --- | --- | --- | --- |
| Jin [1] | 2023 | GLCM | Detection of bony metastases in colorectal cancer |
| Fields [2] | 2021 | GLSZM, LTE, NGTDM, GLRLM, FFT, GLDM, DCT, GLCM | Distinguishing between benign and malignant soft tissue tumors |
| Fields [3] | 2023 | GLSZM, LTE, NGTDM, GLRLM, FFT, GLDM, DCT, GLCM | Predicting soft tissue sarcoma response to chemotherapy |
| Corino [4] | 2018 | GLCM, GLRLM | Distinguishing between intermediate- and high-grade soft tissue sarcomas |
| Gao [5] | 2020 | GLCM, GLRLM, GLSZM, GLDM, NGTDM | Predicting soft tissue sarcoma response to radiotherapy |
| Demirjian [6] | 2022 | LTE, GLSZM, GLRLM, GLDM, GLCM, FFT, DCT | Stratification of tumor grade and stage in renal cell carcinoma |
| Varghese [7] | 2017 | Wavelet transform | Stratifying non-macroscopic fat containing renal masses |
| Jog [8] | 2019 | Curvelet transform | Differentiating clear cell renal cell carcinoma from oncocytoma |
| Naik [9] | 2021 | GLCM, FD | Detection of malignancy in lung nodules |
| Yin [10] | 2020 | GLCM, GLRLM, wavelet transform | Prediction of tumor stage in rectal cancer |
| Li [11] | 2018 | GLCM, GLRLM | Differentiating benign from malignant breast tumors |
| Fan [12] | 2019 | GLCM, GLDM | Differentiating between urothelial and micropapillary carcinoma of the bladder |
| Torheim [13] | 2014 | GLCM | Prediction of relapse in post-treatment cervical cancer |
| Orlhac [14] | 2014 | GLCM, GLRLM, NGTDM, GLZLM | Correlations with SUV_max_, metabolic volumes, and total lesion glycolysis |
| Gomez [15] | 2012 | GLCM | Distinguishing benign from malignant breast lesions |
| Shi [16] | 2010 | Spatial gray-level dependence, FD | Classification of breast lesions |
| Zacharaki [17] | 2009 | Gabor filter | Classification of brain tumor histological subtypes |
| Bader [18] | 2000 | Fourier analysis, GLCM | Differentiating benign from malignant breast lesions |
| Hayano [19] | 2014 | FD | Predicting response to antiangiogenic therapy in hepatocellular carcinoma |
| Lopes [20] | 2011 | FD, GLCM, Gabor filter, wavelet transform | Detection of malignant prostate lesions |
| Tochigi [21] | 2017 | FD | Correlations with metabolic heterogeneity and survival in esophageal cancer |
| Chen [22] | 2022 | GLCM, GLSZM, GLRLM, NGTDM, GLDM | Prediction of occult axillary lymph node metastatic disease in breast cancer |
| Umutlu [23] | 2022 | GLCM, GLRLM, GLSZM, NGLDM, NGTDM | Prediction of response to neoadjuvant chemotherapy in breast cancer |
| Eifer [24] | 2022 | GLCM, GLDM, GLRLM, GLSZM, NGTDM | Differentiating between reactive axillary lymphadenopathy and nodal metastatic breast cancer |
| Chen [25] | 2019 | GLCM, NGLDM, GLZLM, GLRLM | Differentiating between glioblastoma and metastatic brain tumors |
| Lin [26] | 2020 | GLCM, GLSZM, GLRLM, NGTDM | Predicting response to neoadjuvant chemotherapy in osteosarcoma |

GLCM, Gray-Level Co-Occurrence Matrix; GLSZM, Gray-Level Size-Zone Matrix; LTE, Laws Texture Energy; NGTDM, Neighborhood Gray-Tone Difference Matrix; GLRLM, Gray-Level Run-Length Matrix; FFT, Fast Fourier Transform; GLDM, Gray-Level Dependence Matrix; DCT, Discrete Cosine Transform; FD, Fractal Dimension; GLZLM, Gray-Level Zone-Length Matrix; NGLDM, Neighborhood Gray-Level Dependence Matrix

**References**

[1] Jin J, Zhou H, Sun S, Tian Z, Ren H, Feng J, Jiang X. Machine learning based gray-level co-occurrence matrix early warning system enables accurate detection of colorectal cancer pelvic bone metastases on MRI. Front Oncol 2023;13. doi: 10.3389/fonc.2023.1121594

[2] Fields BKK, Demirjian NL, Hwang DH, Varghese BA, Cen SY, Lei X, Desai B, Duddalwar V, Matcuk GR Jr. Whole-tumor 3D volumetric MRI-based radiomics approach for distinguishing between benign and malignant soft tissue tumors. Eur Radiol 2021;31(11):8522-8535. doi: 10.1007/s00330-021-07914-w

[3] Fields BKK, Demirjian NL, Cen SY, Varghese BA, Hwang DH, Lei X, Desai B, Duddalwar V, Matcuk GR Jr. Predicting soft tissue sarcoma response to neoadjuvant chemotherapy using an MRI-based delta-radiomics approach. Mol Imaging Biol 2023. doi: 10.1007/s11307-023-01803-y

[4] Corino VDA, Montin E, Messina A, Casali PG, Gronchi A, Marchiano A, Mainardi LT. Radiomic analysis of soft tissues sarcomas can distinguish intermediate from high-grade lesions. J Magn Reson Imaging 2018;47(3):829-840. doi: 10.1002/jmri.25791

[5] Gao Y, Kalbasi A, Hsu W, Ruan D, Fu J, Shao J, Cao M, Wang C, Eilber FC, Bernthal N, Bukata S, Dry SM, Nelson SD, Kamrava M, Lewis J, Low DA, Steinberg M, Hu P, Yang Y. Treatment effect prediction for sarcoma patients treated with preoperative radiotherapy using radiomics features from longitudinal diffusion-weighted MRIs. Phys Med Biol 2020;65(17):175006. doi: 10.1088/1361-6560/ab9e58

[6] Demirjian NL, Varghese BA, Cen SY, Hwang DH, Aron M, Siddiqui I, Fields BKK, Lei X, Yap FY, Rivas M, Reddy SS, Zahoor H, Liu DH, Desai M, Rhie SK, Gill IS, Duddalwar V. CT-based radiomics stratification of tumor grade and TNM stage of clear cell renal cell carcinoma. Eur Radiol 2022;32(4):2552-2563. doi: 10.1007/s00330-021-08344-4

[7] Varghese B, Hwang D, Mohamed P, Cen S, Deng C, Chang M, Duddalwar V. Wavelets analysis for differentiating solid, non-macroscopic fat containing, enhancing renal masses: a pilot study. Proc SPIE 10572, 13th International Symposium on Medical Information Processing and Analysis, 105720T, November 17, 2017. doi: 10.1117/12.2285948

[8] Jog C, Varghese BA, Hwang DH, Cen SY, Aron M, Desai M, Duddalwar VA. Differentiating clear cell renal cell carcinoma from oncocytoma using curvelet transform analysis of multiphase CT: preliminary study. Proc SPIE 11330, 15th International Symposium on Medical Information Processing and Analysis, 1133009, January 3, 2020. doi: 10.1117/12.2540169

[9] Naik A, Edla DR, Dharavath R. Prediction of Malignancy in Lung Nodules Using Combination of Deep, Fractal, and Gray-Level Co-Occurrence Matrix Features. Big Data 2021;9(6):480-498. doi: 10.1089/big.2020.0190

[10] Yin JD, Song LR, Lu HC, Zheng X. Prediction of different stages of rectal cancer: Texture analysis based on diffusion-weighted images and apparent diffusion coefficient maps. World J Gastroenterol 2020;26(17):2082-2096. doi: 10.3748/wjg.v26.i17.2082

[11] Li Z, Yu L, Wang X, Yu H, Gao Y, Ren Y, Wang G, Zhou X. Diagnostic Performance of Mammographic Texture Analysis in the Differential Diagnosis of Benign and Malignant Breast Tumors. Clin Breast Cancer 2018;18(4):e621-e627. doi: 10.1016/j.clbc.2017.11.004

[12] Fan TW, Malhi H, Varghese B, Cen S, Hwang D, Aron M, Rajarubendra N, Desai M, Duddalwar V. Computed tomography-based texture analysis of bladder cancer: differentiating urothelial carcinoma from micropapillary carcinoma. Abdom Radiol (NY) 2019;44(1):201-208. doi: 10.1007/s00261-018-1694-x

[13] Torheim T, Malinen E, Kvaal K, Lyng H, Indahl UG, Andersen EK, Futsaether CM. Classification of dynamic contrast enhanced MR images of cervical cancers using texture analysis and support vector machines. IEEE Trans Med Imaging 2014;33(8):1648-1656. doi: 10.1109/TMI.2014.2321024

[14] Orlhac F, Soussan M, Maisonobe JA, Garcia CA, Vanderlinden B, Buvat I. Tumor texture analysis in 18F-FDG PET: relationships between texture parameters, histogram indices, standardized uptake values, metabolic volumes, and total lesion glycolysis. J Nucl Med 2014;55(3):414-422. doi: 10.2967/jnumed.113.129858

[15] Gomez W, Pereira WC, Infantosi AF. Analysis of co-occurrence texture statistics as a function of gray-level quantization for classifying breast ultrasound. IEEE Trans Med Imaging 2012;31(10):1889-1899. doi: 10.1109/TMI.2012.2206398

[16] Shi X, Cheng HD, Hu L, Ju W, Tian J. Detection and classification of masses in breast ultrasound images. Digital Signal Processing 2010;20(3):824-836. doi: 10.1016/j.dsp.2009.10.010

[17] Zacharaki EI, Wang S, Chawla S, Soo Yoo D, Wolf R, Melhem ER, Davatzikos C. Classification of brain tumor type and grade using MRI texture and shape in a machine learning scheme. Magn Reson Med 2009;62(6):1609-1618. doi: 10.1002/mrm.22147

[18] Bader W, Bohmer S, van Leeuwen P, Hackmann J, Westhof G, Hatzmann W. Does texture analysis improve breast ultrasound precision? Ultrasound Obstet Gynecol 2000;15(4):311-316. doi: 10.1046/j.1469-0705.2000.00046.x

[19] Hayano K, Lee SH, Yoshida H, Zhu AX, Sahani DV. Fractal analysis of CT perfusion images for evaluation of antiangiogenic treatment and survival in hepatocellular carcinoma. Acad Radiol 2014;21(5):654-660. doi: 10.1016/j.acra.2014.01.020

[20] Lopes R, Ayache A, Makni N, Puech P, Villers A, Mordon S, Betrouni N. Prostate cancer characterization on MR images using fractal features. Med Phys 2011;38(1):83-95. doi: 10.1118/1.3521470

[21] Tochigi T, Shuto K, Kono T, Ohira G, Tohma T, Gunji H, Hayano K, Narushima K, Fujishiro T, Hanaoka T, Akutsu Y, Okazumi S, Matsubara H. Heterogeneity of Glucose Metabolism in Esophageal Cancer Measured by Fractal Analysis of Fluorodeoxyglucose Positron Emission Tomography Image: Correlation between Metabolic Heterogeneity and Survival. Dig Surg 2017;34(3):186-191. doi: 10.1159/000447751

[22] Chen K, Yin G, Xu W. Predictive Value of (18)F-FDG PET/CT-Based Radiomics Model for Occult Axillary Lymph Node Metastasis in Clinically Node-Negative Breast Cancer. Diagnostics (Basel) 2022;12(4). doi: 10.3390/diagnostics12040997

[23] Umutlu L, Kirchner J, Bruckmann NM, Morawitz J, Antoch G, Ting S, Bittner AK, Hoffmann O, Haberle L, Ruckhaberle E, Catalano OA, Chodyla M, Grueneisen J, Quick HH, Fendler WP, Rischpler C, Herrmann K, Gibbs P, Pinker K. Multiparametric (18)F-FDG PET/MRI-Based Radiomics for Prediction of Pathological Complete Response to Neoadjuvant Chemotherapy in Breast Cancer. Cancers (Basel) 2022;14(7). doi: 10.3390/cancers14071727

[24] Eifer M, Pinian H, Klang E, Alhoubani Y, Kanana N, Tau N, Davidson T, Konen E, Catalano OA, Eshet Y, Domachevsky L. FDG PET/CT radiomics as a tool to differentiate between reactive axillary lymphadenopathy following COVID-19 vaccination and metastatic breast cancer axillary lymphadenopathy: a pilot study. Eur Radiol 2022;32(9):5921-5929. doi: 10.1007/s00330-022-08725-3

[25] Chen C, Ou X, Wang J, Guo W, Ma X. Radiomics-Based Machine Learning in Differentiation Between Glioblastoma and Metastatic Brain Tumors. Front Oncol 2019;9:806. doi: 10.3389/fonc.2019.00806

[26] Lin P, Yang PF, Chen S, Shao YY, Xu L, Wu Y, Teng W, Zhou XZ, Li BH, Luo C, Xu LM, Huang M, Niu TY, Ye ZM. A delta-radiomics model for preoperative evaluation of neoadjuvant chemotherapy response in high-grade osteosarcoma. Cancer Imaging 2020;20(1):7. doi: 10.1186/s40644-019-0283-8
